# Supplementary material for: KAT8 compound inhibition inhibits the initial steps of PINK1-dependant mitophagy
Source: Sci Rep. 2024 May 22;14:11721. doi: 10.1038/s41598-024-60602-9 (PMC11111795; doi:10.1038/s41598-024-60602-9)
Supplement: Supplementary file 1 — Supplementary Information 1. [file 41598_2024_60602_MOESM1_ESM.docx]

**Supplementary Figure 1**

**Dose-response of NU9056 and MG149 respectively**

**A.** Quantification of NU9056 dose-response looking at integrated pUb(Ser65) signal intensity, compared to control conditions (n=4, two-way ANOVA with Dunnett’s correction). POE SH-SY5Y cells pre-treated with DMSO, 1µM, 2µM, 4µM, 5µM, 6µM, 8µM, 10µM NU9056 for 3hr and subsequently treated with DMSO or 1µM O/A for 3hr. Cells were then immuno-stained with Hoechst and anti-pUb(Ser65).

**B.** Quantification of MG149 dose-response looking at integrated pUb(Ser65) signal intensity, compared to control conditions (n=4, two-way ANOVA with Dunnett’s correction). POE SH-SY5Y cells pre-treated with DMSO, 1µM, 10µM, 40µM, 50µM, 60µM, 80µM, 90µM, 100µM MG149 for 3hr and subsequently treated with DMSO or 1µM O/A for 3hr. Cells were then immuno-stained with Hoechst and anti-pUb(Ser65).

Data shown as mean +/- SD and significance within DMSO vs O/A treatment groups indicated by *.

**A**

**B**

pUb(Ser65) signal intensity (normalised to control O/A)

pUb(Ser65) signal intensity (normalised to control O/A)

NU9056

MG149
