# Supplementary material for: KAT8 compound inhibition inhibits the initial steps of PINK1-dependant mitophagy
Source: Sci Rep. 2024 May 22;14:11721. doi: 10.1038/s41598-024-60602-9 (PMC11111795; doi:10.1038/s41598-024-60602-9)
Supplement: Supplementary file 2 — Supplementary Information 2. [file 41598_2024_60602_MOESM2_ESM.docx]

**Supplementary Figure 2**

**Characterization of PINK1 KO by western blot**

Representative immunoblots of mitochondrial fractions from POE SH-SY5Y and PINK1-KO POE SH-SY5Y cells treated with DMSO or 1µM O/A for 3hr. Blots were probed for PINK1, pUb(Ser65), Tim23, PDHE1α, and Hsp60.

PINK1 KO

POE

- + - + 1 μM O/A (3hr)

PINK1

kDa


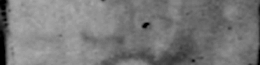


Mitochondrial fraction


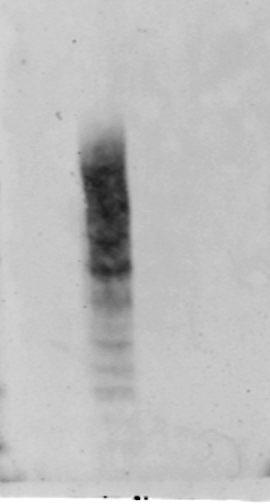


125 –

70 –

pUb

(Ser65)

50 –

15 –

25 –

30 –

38 –

PDHE1α


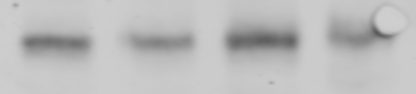


Hsp60

60 –

60 –


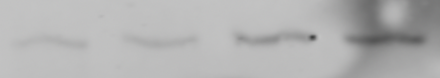


43 –

23 –


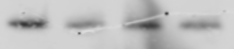


Tim23
