# Supplementary material for: KAT8 compound inhibition inhibits the initial steps of PINK1-dependant mitophagy
Source: Sci Rep. 2024 May 22;14:11721. doi: 10.1038/s41598-024-60602-9 (PMC11111795; doi:10.1038/s41598-024-60602-9)
Supplement: Supplementary file 3 — Supplementary Information 3. [file 41598_2024_60602_MOESM3_ESM.docx]

| SgRNA ID | 5’-3’ sgRNA Sequence (PAM) | 5’-3’ Forward ssDNA Oligo with BbSI Overhang | 5’-3’ Reverse ssDNA Oligo with BbSI Overhang |
| --- | --- | --- | --- |
| PINK1_Seq1 | CGCCACCATGGCGGTGCGAC(AGG) | caccgCGCCACCATGGCGGTGCGAC | aaacGTCGCACCGCCATGGTGGCGc |
| PINK1_Seq2 | ACCGGGCGCGGAGCCTCGCA(GGG) | caccgACCGGGCGCGGAGCCTCGCA | aaacTGCGAGGCTCCGCGCCCGGTc |

**Supplementary Table 1**

**5’-3’ Forward and Reverse ssDNA sequences**
